# The Impact of Asthma Control on the Clinical and Depressive Symptoms in Pediatric Asthma Patients

**DOI:** 10.62641/aep.v53i3.1996

**Published:** 2025-05-05

**Authors:** Yunfang Li, Wenwen Guo, Xiaoqing Li, Xiaoqing Su, Shuyun Zheng, Xianfeng Qu

**Affiliations:** ^1^Department of Pediatrics, Jiaozhou Central Hospital of Qingdao, 266300 Qingdao, Shandong, China; ^2^Endoscopy Center, Jinan Zhangqiu District People’s Hospital, 250200 Jinan, Shandong, China; ^3^Department of Pediatrics, Jinan Zhangqiu District People’s Hospital, 250200 Jinan, Shandong, China; ^4^Infectious Disease Department, Qingdao Women and Children’s Hospital, 266000 Qingdao, Shandong, China

**Keywords:** asthma, depression, child, healthy, quality of life

## Abstract

**Background:**

Asthma is a common chronic respiratory disease that severely affects children’s health and leads to anxiety and depressive symptoms. Therefore, this study aims to investigate the correlation between asthma control status and depressive symptoms in children with asthma.

**Methods:**

This study included pediatric asthma patients (n = 117) who were admitted to Jiaozhou Central Hospital of Qingdao between January 2021 and January 2023. Based on asthma control status, the patients were divided into well-controlled (n = 67) and poorly controlled (n = 50) groups. Various parameters, including asthma control, depressive symptoms, respiratory function, sleep quality, physical activity, and quality of life, were assessed through standardized assessments, medical records, and participant, or caregiver reports. The data was comparatively analyzed between the two groups.

**Results:**

We observed no significant differences in baseline characteristics between the two experimental groups (*p* > 0.05). Furthermore, the well-controlled group demonstrated better asthma control with lower hospitalizations, emergency visits, and higher asthma control test scores (*p* < 0.001). Additionally, the well-controlled group exhibited better respiratory function with higher forced vital capacity (FVC), better ratio of forced expiratory volume in one second to forced vital capacity, higher maximal mid-expiratory flow, higher forced expiratory flow between 25%–75% of FVC, and higher total lung capacity (*p* < 0.001). Moreover, the symptom score (*p* < 0.001), activity restriction score (*p* = 0.001), and affective function score (*p* < 0.001) were significantly higher for the well-controlled group compared to the poorly controlled group. Additionally, the well-controlled group showed lower levels of childhood depression, as evidenced by lower Children’s Depression Inventory scores (*p* < 0.001).

**Conclusion:**

Our findings provide strong evidence of the association between asthma control status and childhood depressive symptoms in pediatric asthma patients. Effective asthma management was associated with lower levels of depressive symptoms, better respiratory function, improved sleep quality, increased physical activity, and higher quality of life, highlighting the need for comprehensive care and integrated management approaches in pediatric asthma to optimize health outcomes. These findings hold significance for clinical practice, offering valuable insights into the subtle factors influencing asthma control and depressive symptoms in pediatric asthma patients.

## Introduction

Asthma is a prevalent chronic respiratory condition in children, characterized by recurrent episodes of wheezing, breathlessness, chest tightness, and coughing, often triggered by allergens, physical activity, or respiratory infections [1, 2, 3, 4]. Globally, asthma affects about 339 million individuals, with a growing prevalence rate, particularly among children [5, 6, 7]. The burden of pediatric asthma extends beyond the physical manifestations, impacting daily life and adversely affecting psychological and emotional well-being [8, 9, 10].

Childhood depression is a major mental health concern that can significantly affect a child’s overall quality of life and future outcomes [11, 12]. Pediatric depression has been associated with impaired social functioning, lower academic performance, and an increased risk of developing lifelong mental health disorders [13, 14]. While the relationship between asthma and depression has been recognized in clinical settings, most of the previous research have primarily focused on the prevalence of depressive symptoms in individuals with asthma and the potential impact of these symptoms on asthma outcomes [15, 16, 17, 18]. Studies have revealed a higher prevalence of depressive symptoms among individuals with asthma compared to the general population, and have highlighted bidirectional the relationship, where asthma can exacerbate depressive symptoms and vice versa [19, 20, 21]. Moreover, depressive symptoms among individuals with asthma have been correlated with poorer asthma control, reduced adherence to asthma medication, and increased healthcare utilization, emphasizing the complex implications of mental health on the management of asthma [22, 23, 24]. Furthermore, beyond depressive symptoms, the psychological burden of asthma also impacts emotional well-being, anxiety levels, and quality of life [25, 26, 27]. Children with asthma often experience elevated emotional distress related to their condition, challenges in social interactions, and limitations in daily activities, all of which can contribute to psychological stress and affect their overall well-being [28, 29]. Despite these insights, research directly addressing the correlation between asthma control and depressive symptoms in pediatric asthma patients remains limited.

Despite advancements in asthma management and treatment, a substantial number of children with asthma continue to experience suboptimal disease control, characterized by recurrent symptoms, exacerbations, and impaired lung function. Poor asthma control not only increases healthcare utilization and medication requirements but may also elevate psychological burden, potentially impacting the mental health of these children.

Understanding the association between asthma control and childhood depressive symptoms is essential for optimizing comprehensive care for pediatric asthma patients. By elucidating this link, healthcare providers can implement targeted interventions that address both the physical and emotional aspects of asthma, thereby enhancing overall disease management and improving the well-being of patients. Therefore, this retrospective cohort study aims to investigate the correlation between asthma control and childhood depressive symptoms in pediatric asthma patients.

## Materials and Methods

### Study Participants

This retrospective cohort study included pediatric patients diagnosed with asthma who were admitted to Jiaozhou Central Hospital of Qingdao between January 2021 and January 2023. Based on asthma control, the participants were divided into two groups: the well-controlled group (n = 67) and the poorly controlled group (n = 50). This study was approved by the Medical Ethics Committee of Jiaozhou Central Hospital of Qingdao (Approval No.: 2023410302) and adhered to the declaration of Helsinki guidelines. The Institutional Review Board and Ethics Committee of Jiaozhou Central Hospital of Qingdao waived the need for informed consent, as it exclusively used de-identified patient data, ensuring no risk or impact on patient care.

### Grouping Criteria of the Patients

The level of disease control among the children was monitored using the Asthma Symptom Scoring Scale [30], which includes daytime and nighttime symptom scores. Daytime symptom scoring criteria were as follows: 0 points indicated no coughing, wheezing, chest tightness, or breathing difficulties; 1 point indicated mild or intermittent occurrence of these symptoms; 2 points indicated the symptoms being relatively severe or frequent; 3 points indicated persistent symptoms, affecting daily life. For nighttime symptom scoring, 0 points indicated no symptoms during sleep; 1 point indicated awakening once; 2 points represented awakening twice or more; 3 points indicated frequent awakening during sleep that significantly impacted sleep quality. Patients with scores of 3 or higher were classified as the poorly controlled group, while those scores below 3 were categorized as the well-controlled group.

### Inclusion and Exclusion Criteria

Inclusion criteria included, pediatric patients diagnosed with asthma, those with ages ranging from 7–17 years, and those with mild to moderate disease. However, exclusion criteria for the patients were as follows: Patients with a history of other chronic respiratory conditions, such as chronic obstructive pulmonary disease (COPD) or cystic fibrosis; patients with significant comorbidities (e.g., cardiac conditions, severe neurological disorders) that could impact the assessment of asthma control, respiratory function, or mental health outcomes; those with incomplete or insufficient medical records, including missing data on asthma control parameters, depression scores, or relevant clinical assessments; patients with a history of psychiatric disorders other than depression, which could confound the assessment of childhood depression; and those with cognitive or developmental impairments that could affect their ability to self-report symptoms or participate in mental health and quality of life assessments.

### Asthma Treatment Method

The children received montelukast (produced by Lunan Better Pharmaceutical Co., Ltd., Shandong, China, with National Medicine Number H20083372) at a dose of 5 mg/day after dinner for 8 weeks.

### Children’s Depression Inventory Score

The Children’s Depression Inventory (CDI) score was utilized to evaluate depressive symptoms among patients. This is a widely used self-report assessment tool for measuring depressive symptoms in children and adolescents (aged 7 to 17). It consists of 27 items that assess various aspects of depressive symptoms, such as negative mood, interpersonal problems, ineffectiveness, anhedonia, and negative self-esteem. Each item is scored on a scale of 0 to 2, with higher scores indicating more severe depressive symptoms. The CDI score has shown strong reliability and validity in clinical and nonclinical cohorts, with a Cronbach’s α of 0.86 [31].

### Quality of Life Assessment

The patient’s quality of life was evaluated using the Pediatric Asthma Quality of Life Questionnaire (PAQLQ) [32]. This questionnaire covers three dimensions: symptoms (10 questions), activity limitation (5 questions), and emotional function (8 questions). The total score is determined as the average of the scores from these three dimensions (ranging from 1 to 7), with higher scores indicating better quality of life [33, 34].

### Data Collection

Data on baseline characteristics, asthma control, depressive symptoms, respiratory function, and quality of life were collected through standardized assessments, medical records, and participant or caregiver reports. The data related to scale evaluation were collected by the medical staff of Jiaozhou Central Hospital of Qingdao and assessed by the attending physician. The data collection period was 1 year. Mean scores for asthma control, quality of life, and the Children’s Depression Inventory were included in the analysis.

### Statistical Analysis

The general characteristics, asthma control, depressive symptoms, respiratory function, quality of life, and asthma symptoms were comparatively analyzed between the two patient groups using SPSS 25.0 (International Business Machines Corporation, Armonk, NY, USA). Frequency data are presented as n (%), and chi-square tests were conducted for sample sizes of ≥40 with theoretical frequencies (T) of ≥5 or more. For sample sizes ≥40, where 1 ≤ T < 5, a corrected formula for the chi-square test was used. If the sample size was <40 or T <1, Fisher’s exact probability method was employed for statistical analysis. The Shapiro-Wilk method was employed to determine the normality in continuous variables when comparing the two groups. For normally distributed continuous data, results are expressed as mean ± standard deviation using the *t*-test, while the data not conforming to the normal distribution were represented as [M (P25, P75)], and analyzed using the Mann-Whitney U test.

## Results

### Comparison of Baseline Characteristics Between the Two Groups

Among the 117 study participants, 67 were classified as well-controlled asthma and 50 as poorly controlled asthma. There was no significant difference between the two groups (*p* > 0.05). Both groups had a majority of male patients with comparable body mass index (BMI). The maternal education levels were higher in the well-controlled group than in the poorly controlled group, although this difference was not statistically significant (*p* > 0.05). Furthermore, the well-controlled group had a shorter duration of asthma compared to the poorly controlled group (*p* > 0.05). The average drug use was slightly lower in the well-controlled group compared to the poorly controlled group, with no significant difference found (*p* > 0.05). Furthermore, the average duration of medication was slightly lower in the well-controlled group than the poorly controlled group, with no significant difference observed (*p* > 0.05). Similarly, there were no significant differences between the two groups regarding socioeconomic status, mode of delivery, or medication-related characteristics (Table 1).

**Table 1.** **Comparison of baseline characteristics between the two groups**.

Characteristic		Well-controlled group (n = 67)	Poorly controlled group (n = 50)	z/*t*/χ^2^	*p*-value
Age (years)		8.00 (7.00, 9.00)	8.00 (7.00, 9.00)	–1.338	0.181
Gender (M/F)		34/33	26/24	0.018	0.893
BMI (kg/m^2^)		20.44 ± 2.17	20.82 ± 1.16	–1.132	0.260
Maternal education (years)		13.60 ± 2.63	13.29 ± 2.74	0.617	0.538
Duration of asthma (years)		3.56 ± 1.13	3.87 ± 1.34	–1.378	0.171
Socioeconomic status		3.82 ± 0.49	3.67 ± 0.75	1.307	0.194
Mode of delivery				0.034	0.853
	Vaginal delivery	36 (53.73%)	26 (52.00%)		
	Cesarean section	31 (46.27%)	24 (48.00%)		
	Drug use (mg/day)	4.92 ± 0.74	5.04 ± 0.77	–0.892	0.374
	Duration of drug use (days)	52.99 ± 2.51	53.36 ± 2.35	–0.827	0.410

BMI, body mass index.

### Comparison of Asthma Control Between the Two Groups

The well-controlled asthma group demonstrated significantly lower hospitalizations in the past year compared to the poorly controlled group (*p* < 0.001) (Table 2). Similarly, emergency visits in the past year were significantly lower in the well-controlled group than in the poorly controlled group (*p* < 0.001). Furthermore, asthma control test scores were markedly higher in the well-controlled group than in the poorly controlled group (*p* < 0.001). These findings indicate a clear trend of better asthma control in the well-controlled group across all parameters, highlighting the potential impact of effective asthma management on healthcare utilization and disease control.

**Table 2.** **Comparison of asthma control between the two groups**.

Characteristic	Well-controlled group (n = 67)	Poorly controlled group (n = 50)	*t*	*p*-value
Hospitalizations (past year)	1.45 ± 0.59	3.26 ± 1.16	10.970	<0.001
Emergency visits (past year)	2.69 ± 0.95	4.93 ± 1.88	–8.468	<0.001
Asthma control test score	23.51 ± 3.90	16.48 ± 3.11	10.485	<0.001

### Comparison of Respiratory Function Test Results Between the Two Groups

Comparing respiratory function test results between the two groups showed significant differences across multiple parameters (Table 3). The well-controlled group exhibited higher forced vital capacity (FVC) than the poorly controlled group (*p* < 0.001). Additionally, the well-controlled group demonstrated a higher ratio of forced expiratory volume in one second to forced vital capacity (FEV1/FVC) than the poorly controlled group (*p* < 0.001). Moreover, maximal mid-expiratory flow (MMEF), forced expiratory flow between 25%–75% of FVC (FEF25–75%), and total lung capacity (TLC) were also significantly higher in the well-controlled group compared to the poorly controlled group (*p* < 0.001). These findings indicate a better respiratory function in the well-controlled asthma group, underscoring the potential impact of effective asthma control on pulmonary function.

**Table 3.** **Comparison of respiratory function test results between the two groups of patients**.

Characteristic	Well-controlled group (n = 67)	Poorly controlled group (n = 50)	*t*	*p*-value
FVC (L)	2.56 ± 0.50	2.16 ± 0.49	4.357	<0.001
FEV1/FVC	0.84 ± 0.03	0.78 ± 0.04	9.497	<0.001
MMEF (L/s)	4.61 ± 0.73	3.20 ± 0.84	9.661	<0.001
FEF25–75% (L/s)	3.37 ± 0.59	2.06 ± 0.64	11.423	<0.001
TLC (L)	4.39 ± 0.47	3.61 ± 0.54	8.252	<0.001

FVC, forced vital capacity; FEV1, forced expiratory volume in one second; MMEF, maximal mid-expiratory flow; FEF25–75%, forced expiratory flow between 25%–75% of FVC; TLC, total lung capacity.

### Comparison of Quality of Life Between the Two Groups (PAQLQ)

The comparison of quality of life, as measured by the PAQLQ, between the two groups revealed significant differences across all domains (Table 4). The symptom score (*p* < 0.001), activity restriction score (*p* = 0.001), and affective function score (*p* < 0.001) were significantly higher for the well-controlled group compared to the poorly controlled group. These findings suggest a significant association between effective asthma control and improved quality of life among pediatric patients, highlighting the positive impacts of optimal asthma management on all aspects of daily functioning and emotional health.

**Table 4.** **Comparison of quality of life between the two groups (PAQLQ)**.

	Symptom score	Activity restriction score	Affective function score
Well-controlled group (n = 67)	4.91 ± 0.55	3.72 ± 0.64	6.13 ± 0.38
Poorly controlled group (n = 50)	3.07 ± 0.43	3.35 ± 0.52	4.21 ± 0.42
*t*	19.586	3.330	25.658
*p*-value	<0.001	0.001	<0.001

PAQLQ, Pediatric Asthma Quality of Life Questionnaire.

### Comparison of Depressive Symptoms Between the Two Groups

The comparison of depressive symptoms between the two groups revealed significant differences (Table 5). The Children’s Depression Inventory (CDI) score was significantly lower in the well-controlled asthma group than in the poorly controlled group (*p* < 0.001), indicating lower levels of depressive symptoms in the well-controlled group. These findings suggest a potential association between asthma control and lower levels of depressive symptoms, highlighting the potential impact of asthma control on mental health outcomes.

**Table 5.** **Comparison of depressive symptoms between the two groups**.

Parameter	Well-controlled group (n = 67)	Poorly controlled group (n = 50)	*t*	*p*-value
Children’s Depression Inventory (CDI) score	13.37 ± 3.46	19.74 ± 4.29	–8.886	<0.001

## Discussion

The findings from this retrospective cohort study elucidate the multifaceted implications of effective asthma management on various aspects of pediatric well-being, including respiratory function, sleep quality, physical activity, and quality of life. Furthermore, the study provides compelling evidence for the potential association between asthma control status and childhood depressive symptoms, highlighting the need for comprehensive care to enhance health outcomes for children with asthma.

The differences in asthma control status between the well-controlled and poorly controlled groups underscore the impact of effective asthma management on various health parameters. Notably, the well-controlled asthma group exhibited lower rates of hospitalizations and emergency visits, indicating a reduced disease burden and better disease control. These findings align with a previous study by Shipp CL *et al*. [10], which highlights the significance of optimal asthma control in reducing healthcare utilization and improving disease outcomes. Additionally, the better respiratory function observed in the well-controlled group, as evidenced by higher values for FVC, FEV1/FVC ratio, MMEF, FEF25–75%, and TLC, further emphasizes the potential benefits of effective asthma management in maintaining pulmonary function and overall respiratory health.

Moreover, the study findings reveal a strong association between asthma control status and mental health outcomes, particularly childhood depressive symptoms [35, 36, 37]. The well-controlled asthma group demonstrated lower levels of depressive symptoms, as indicated by their lower scores on the Children’s Depression Inventory. These results suggest that effective asthma control may offer a protective effect on children’s mental health, potentially reducing the risk of developing depressive symptoms.

Furthermore, the study underscores the broader impact of asthma control on the overall quality of life of children. The well-controlled group reported a better quality of life across various domains, highlighting the extensive implications of effective asthma control on children’s daily functioning, emotional well-being, and overall quality of life.

Furthermore, our findings signify implications for clinical practice and the comprehensive care of pediatric asthma patients. Understanding the association between asthma control status and childhood depressive symptoms is essential for optimizing comprehensive care for these patients. By elucidating this relationship, healthcare providers can implement targeted interventions to address both the physical and emotional aspects of asthma, ultimately enhancing overall disease management and improving the well-being of affected children. The observed association between asthma control status and mental health outcomes highlights the need for integrated care approaches that consider both the physical and psychological aspects of asthma in pediatric patients. This reinforces the significance of a multidisciplinary approach to pediatric asthma care, which should incorporate mental health screening, psychological support, and tailored interventions to address the broader impact of asthma on children’s well-being.

Moreover, the association between asthma control status and various health parameters, including respiratory function and quality of life, highlights the holistic impact of asthma on pediatric well-being. These findings underscore the need for comprehensive asthma management strategies that address not only the physical manifestations of the condition but also the broader impact on daily functioning, emotional well-being, and overall quality of life. By considering the complex implications of asthma, healthcare providers can develop tailored management plans that meet the individual needs of pediatric patients, ultimately optimizing health outcomes and enhancing overall well-being.

We emphasized the clinical significance of asthma control as a therapeutic approach and its role in improving the health and depressive symptoms of children with asthma. These findings have important implications for clinical practice, offering valuable insights into the subtle factors influencing asthma control and depressive symptoms in pediatric asthma patients. By elucidating the relationships between asthma control and depressive symptoms, respiratory function, sleep quality, physical activity, and quality of life, this comprehensive analysis provides practical guidance for clinicians in managing pediatric asthma patients. The strong correlations observed between asthma control and various clinical parameters underscore the significance of asthma control in guiding treatment plans, resource allocation, and risk stratification.

While the findings of this study provide valuable insights into the relationship between asthma control status and childhood depressive symptoms, several limitations should be addressed. The retrospective nature of this study may limit the establishment of causal relationships, and the relatively small sample size may hinder the generalizability of the findings to diverse populations. Future prospective studies with larger and more diverse cohorts are warranted to further elucidate the relationship between asthma control and mental health outcomes in pediatric patients. Additionally, longitudinal studies could provide valuable insights into the long-term effects of effective asthma management on mental health outcomes and overall well-being in children with asthma.

## Conclusion

In conclusion, these findings offer strong evidence of the association between asthma control status and childhood depressive symptoms in pediatric asthma patients. The differences observed in asthma control status between well-controlled and poorly controlled groups highlight the complex implications of effective asthma management on various aspects of pediatric well-being. These findings underscore the need for comprehensive care approaches to address both the physical and emotional aspects of asthma, emphasizing the importance of integrating mental health considerations into pediatric asthma management. By understanding the interplay between asthma control and mental health outcomes, healthcare providers can optimize holistic care for pediatric asthma patients, thereby improving disease management and overall well-being.

## Availability of Data and Materials

The datasets used and/or analyzed during the current study are available from the corresponding author on reasonable request.
